# L-Alanine Exporter, AlaE, of *Escherichia coli* Functions as a Safety Valve to Enhance Survival under Feast Conditions

**DOI:** 10.3390/ijms20194942

**Published:** 2019-10-07

**Authors:** Satoshi Katsube, Tasuke Ando, Hiroshi Yoneyama

**Affiliations:** Laboratory of Animal Microbiology, Department of Microbial Biotechnology, Graduate School of Agricultural Science, Tohoku University, 468-1, Aramaki Aza Aoba, Aoba-ku, Sendai 980-8572, Japan; tasuke.ando.d4@tohoku.ac.jp (T.A.); hiroshi.yoneyama.a4@tohoku.ac.jp (H.Y.)

**Keywords:** *E. coli*, Alanine, AlaE

## Abstract

The intracellular level of amino acids is determined by the balance between their anabolic and catabolic pathways. L-alanine is anabolized by three L-alanine synthesizing enzymes and catabolized by two racemases and D-amino acid dehydrogenase (DadA). In addition, its level is regulated by L-alanine movement across the inner membrane. We identified the novel gene *alaE*, encoding an L-alanine exporter. To elucidate the physiological function of L-Alanine exporter, AlaE, we determined the susceptibility of *alaE*-, *dadA*-, and *alaE*/*dadA*-deficient mutants, derived from the wild-type strain MG1655, to L-alanyl-L-alanine (Ala-Ala), which shows toxicity to the L-alanine-nonmetabolizing variant lacking *alaE*. The *dadA*-deficient mutant has a similar minimum inhibitory concentration (MIC) (>1.25 mg/mL) to that observed in MG1655. However, *alaE*- and *alaE*/*dadA*-deficient mutants had MICs of 0.04 and 0.0025 mg/mL, respectively. The results suggested that the efficacy of AlaE to relieve stress caused by toxic intracellular accumulation of L-alanine was higher than that of DadA. Consistent with this, the intracellular level of alanine in the *alaE*-mutant was much higher than that in MG1655 and the *dadA*-mutant. We, therefore, conclude that AlaE functions as a ‘safety-valve’ to prevent the toxic level accumulation of intracellular L-alanine under a peptide-rich environment, such as within the animal intestine.

## 1. Introduction

Bacteria live in environments where nutritional conditions change extensively (both temporally and spatially), such as within the animal intestine. To cope with extracellular nutritional fluctuation, maintenance of intracellular metabolic homeostasis involving metabolites, such as amino acids, is important. The intracellular levels of metabolites are, in principle, a balance of anabolic and catabolic metabolisms [1]. In addition, the movement of solutes across the cytoplasmic membrane contributes to their intracellular homeostasis [2]. Amino acid importers are well characterized; expression levels of which are high and low in the absence and presence of amino acids, respectively [3,4]. The regulation of expression of these amino acid importers can be a mechanism to maintain intracellular levels of their substrates within a certain range, besides the anabolic and catabolic pathways of the relevant amino acid. Therefore, the physiological function of amino acid importers is explicit, that is, obtaining building blocks of proteins and metabolic sources of carbon and energy, and the nitrogen source as well [5,6]. However, the function of amino acid exporters is still unclear.

Recently, several amino acid exporters have been identified in *Corynebacterium glutamicum* [7,8,9,10] and *Escherichia coli* [11,12,13,14,15,16,17,18,19,20,21,22,23]. Their functional analyses show that, apart from the mechanosensitive channel for glutamic acid [9], they utilize biological energy to export their substrates, primary metabolites [10,13,14]. This finding poses the enigmatic question of why bacteria possess exporters for the important primary metabolites, i.e., amino acids.

In terms of alanine metabolism in *E. coli*, this bacterium has three major aminotransferases (AvtA, YfbQ, and YfdZ) that synthesize L-alanine from pyruvate [24], two alanine racemases (Alr and DadX) that interconvert alanine enantiomers [25], and D-amino acid dehydrogenase (DadA) that generates pyruvate from D-alanine [26]. In addition to these alanine metabolic pathways, we recently identified an L-alanine exporter, AlaE, by employing an L-alanine non-metabolizing variant, which lacks genes encoding three aminotransferases and two alanine racemases [27]. Furthermore, the expression of the *alaE* gene has been found to be regulated by the global regulator Lrp (leucine responsive protein) in a positive manner in the presence of L-alanine and L-leucine, but not D-alanine [28], suggesting that AlaE could function as a “safety valve” to prevent an abrupt increase of an intracellular L-alanine level that could lead to growth inhibition when *E. coli* faces a feast environmental condition. Still, this hypothesis is not conclusive because (i) the L-alanine non-metabolizing strain used in the previous studies [27,28,29] does not exist in the natural environment and (ii) the alanine catabolic pathway involving DadA has not been taken into account in the earlier studies.

In this study, we further investigated the physiological function of AlaE by comparing the impact of its capacity to relieve stress caused by high accumulation of intracellular L-alanine with that of DadA, by employing isogenic *alaE*-, *dadA*-, and *alaE*/*dadA*-deficient mutants, derived from the wild-type strain MG1655.

## 2. Results and Discussion

### 2.1. Susceptibility of MG1655 and Its Derivatives to L-Alanyl-L-Alanine (Ala-Ala)

To evaluate the impact of a dysfunction of the major L-alanine exporter (AlaE) and the alanine catabolic enzyme, D-amino acid dehydrogenase (DadA), on the metabolism of L-alanine in intact cells, we measured the minimum inhibitory concentration (MIC) of Ala-Ala against the isogenic single mutants, MG1655∆*alaE* and MG1655∆*dadA*, as well as a double mutant, MG1655∆*alaE*∆*dadA* (Table 1). The MIC of Ala-Ala for MG1655∆*dadA* was >1.2 mg/mL, which was the same as that for MG1655 and MLA301, suggesting that an abrupt increase in intracellular L-alanine, derived from Ala-Ala, was prevented by AlaE-mediated L-alanine export.

In contrast, the double mutant MG1655∆*alaE*∆*dadA* was strikingly susceptible to Ala-Ala (MIC, 0.0025 mg/mL), the level of which was the same as that of MLA301∆*alaE*, which completely lacks both L-alanine synthetic and alanine racemase activities [27]. This result was consistent with higher intracellular L-alanine levels in MG1655∆*alaE*∆*dadA* and MLA301∆*alaE* compared to that in MG1655 described below. This result suggested that L-alanine export activity plays a pivotal role in maintaining the intracellular level of L-alanine, under high external L-alanine conditions as suggested by Kim [29] and Hori [27], who assumed a physiological role of AlaE to be that of a ‘safety-valve’. If this hypothesis is the case, the deletion of AlaE is expected to impact the susceptibility of the *alaE*-deficient mutant toward Ala-Ala. Indeed, the MIC for MG1655∆*alaE* was 0.04 mg/mL, which was much lower than that observed for its parent strain MG1655, clearly indicating that AlaE plays the most important role in reducing intracellular L-alanine level under the conditions tested.

Notably, MG1655∆*alaE* was more resistant to Ala-Ala compared to the double mutant MG1655∆*alaE*∆*dadA*, implying that the L-alanine catabolic pathway, via alanine racemases (Alr and DadX), which generate the substrate of D-amino acid dehydrogenase, could contribute to the prevention of intracellular accumulation of L-alanine, to some extent. However, the efficacy of D-amino acid dehydrogenase to limit L-alanine accumulation in the cells must be secondary, since the MIC of Ala-Ala against MG1655∆*dadA* and MG1655 was the same (>1.2 mg/mL).

We next addressed the issue of whether the increased activity of D-amino acid dehydrogenase could lower the stress caused by intracellular L-alanine accumulation by culturing MG1655∆*alaE* in minimal medium containing glycerol as a sole carbon source, which relieves catabolite repression, resulting in the increased expression of the D-amino acid dehydrogenase gene [26]. The susceptibility of MG1655∆*alaE* grown in glycerol medium to Ala-Ala was lower (MIC, 0.16 mg/mL) than that obtained for cells grown in glucose medium (0.04 mg/mL), indicating that D-amino acid dehydrogenase indeed has an ability to relieve an excess accumulation of intracellular L-alanine, to some extent. Still, the enzymatic ability to control the intracellular L-alanine level seemed weaker than that of AlaE, consistent with the intracellular alanine accumulation in various strains, as described below.

It is interesting to note that MLA301∆*alaE* showed much lower MIC (0.0025 mg/mL) of Ala-Ala (high susceptibility) than MG1655∆*alaE* did (0.04 mg/mL). One may argue that MLA301∆*alaE* imports Ala-Ala more rapidly than MG1655∆*alaE*, thereby resulting in higher accumulation of intracellular alanine in MLA301∆*alaE* compared to that in MG1655∆*alaE*. However, this hypothesis is not the case, because MLA301 was derived from MG1655 by deletion of two alanine racemase genes (*alr*, *dadX*) and three aminotransferase genes (*avtA*, *yfbQ*, and *yfdZ*) using the gene replacement technique [20]. Thus, the Ala-Ala uptake activity of MLA301∆*alaE* is theoretically the same as that of MG1655∆*alaE*.

### 2.2. Accumulation of Intracellular Alanine in MG1655 and its Derivatives

**Figure 1:**
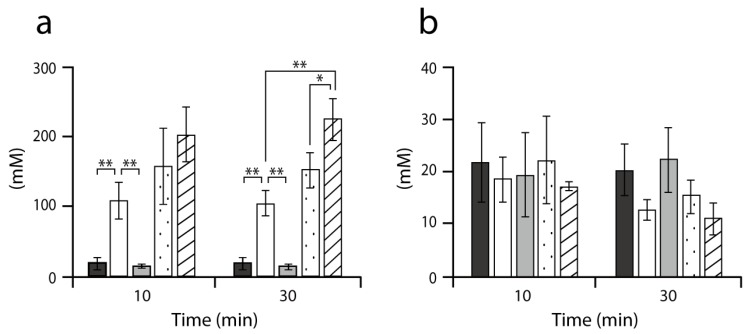
Intracellular accumulation of alanine (**a**) and glutamic acid (**b**). After incubation of strains MG1655 (black), MG1655∆*alaE* (white), MG1655∆*dadA* (grey), MG1655∆*dadA*∆*alaE* (stippled), and MLA301∆*alaE* (hatched) in minimal medium containing 6 mM Ala-Ala, intracellular fractions were recovered by the silicone oil method, as described in materials and methods. Intracellular alanine and glutamic acid were determined by HPLC. Data are means ± standard deviations from three separate experiments. Asterisks indicate statistically significant differences (unpaired Student *t*-test, ** < 0.01, * < 0.05).

We found that AlaE has a primary role in maintaining the intracellular level of L-alanine, the function of which was complemented by D-amino acid dehydrogenase. To substantiate this, we next determined intracellular alanine levels in several isogenic mutants in the presence of 6 mM Ala-Ala (Figure 1a). The level of alanine in MG1655∆*alaE* was approximately 100 mM, which was about 5-fold higher than in MG1655. This result was consistent with the above Ala-Ala susceptibility test (Table 1) and with our previous studies [27,29]. In contrast, MG1655∆*dadA* accumulated alanine in the cells to a similar level as the wild-type strain, implying that the activity of AlaE, but not DadA, mainly contributes to the reduction of intracellular alanine. In addition, the intracellular level of alanine in MLA301 [27], which has a normal L-alanine export activity, was comparable to that in MG1655 (Figure 1). These results indicated again that AlaE is the primary route to circumvent the intracellular accumulation of alanine under the conditions tested. 

Furthermore, the intracellular alanine level in MG1655∆*alaE* was marginally lower than that observed in the double mutant MG1655∆*alaE*∆*dadA*. This result was interpreted to mean that, in MG1655∆*alaE*, D-amino acid dehydrogenase catabolizes D-alanine to generate pyruvic acid, leading to a reduction of intracellular D-alanine. In turn, L-alanine is transformed into D-alanine by alanine racemases, which eventually resulted in the reduction of alanine to the level observed in MG1655∆*alaE*. 

Interestingly, the intracellular alanine level in MLA301∆*alaE*, in which L-alanine anabolic and catabolic activities were nullified, tended to be slightly higher than that observed in MG1655∆*alaE*∆*dadA*. With this result, one may argue that MG1655∆*alaE*∆*dadA* may have another route(s) that lowers the intracellular alanine level. We could presume four possibilities: (i) a reverse reaction of L-alanine biosynthetic enzymes, alanine aminotransferases (AvtA, YfbQ, and YfdZ), that generates L-alanine from pyruvate, (ii) the peptidoglycan synthesis via alanine racemase reaction, (iii) higher Ala-Ala import activity in MLA301∆*alaE* compared to that in MG1655∆*alaE*∆*dadA*, or (iv) D-alanine excretion, that, in turn, leads to the reduction of intracellular alanine level in MG1655∆*alaE*∆*dadA*. If the first possibility was correct, one could expect that: (i) glutamic acid would be formed, resulting in accumulation of glutamic acid in the cells and (ii) the MIC of Ala-Ala for MG1655∆*alaE*∆*dadA* would be higher than that observed in MLA301∆*alaE*, due to the conversion of L-alanine to pyruvic acid. However, this is not the case, since the level of intracellular glutamic acid in MG1655∆*alaE*∆*dadA* was similar to that in MLA301∆*alaE* (Figure 1b), and MG1655∆*alaE*∆*dadA* and MLA301∆*alaE* had the same MIC of Ala-Ala (Table 1). In addition, this result was consistent with a previous observation that a D-amino acid dehydrogenase-deficient mutant could not utilize L-alanine and D-alanine as its sole carbon source [25]. The second scenario is improbable, as the amount of D-alanine incorporated in peptidoglycan cannot explain the reduction of intracellular alanine levels observed under these test conditions [1]. The third possibility is very unlikely because, as described previously, both strains are isogenic. With regard to the last possibility, *E. coli* has recently been found to possess a system(s) that secretes D-alanine [30], an observation that correlates well with the lower alanine level in MG1655∆*alaE*∆*dadA* as compared to that in MLA301∆*alaE* (Figure 1b).

### 2.3. Coculture Assay in the Presence of Ala-Ala

**Figure 2:**
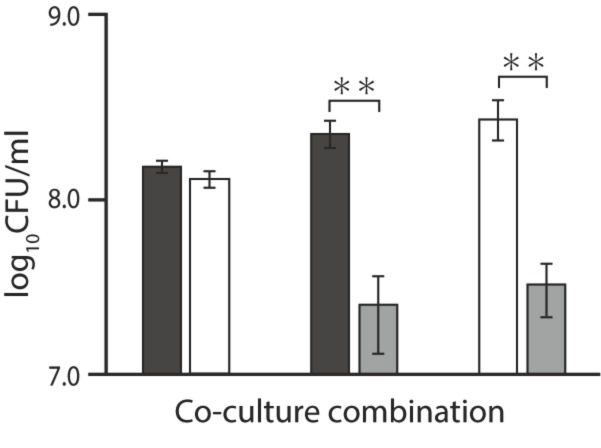
Survival of *alaE*- and *dadA*-deficient mutants in the presence of Ala-Ala in a coculture system. Three pairs of MG1655/MG1655∆*dadA*, MG1655/MG1655∆*alaE*, and MG1655∆*dadA*/MG1655∆*alaE* harboring pBR322 or pBR325 were cocultured in minimal medium in the presence of 5 mM Ala-Ala. When cultures reached a mid-log phase, colony-forming units of each strain were determined. Black, white, and grey bars indicate MG1655, MG1655∆*dadA*, and MG1655∆*alaE*, respectively. Data presented are the means ± standard deviations from three separate experiments. Asterisks indicate statistically significant differences (unpaired Student *t*-test, ** < 0.01).

From the above results, we assumed that L-alanine exporter AlaE plays the most critical role in avoiding a toxic accumulation of L-alanine under high L-alanine conditions. If this hypothesis holds, AlaE could confer a survival advantage compared to AlaE-deficient cells, under conditions where the intracellular alanine level abruptly increases. To test this, we determined the survival of isogenic mutants MG1655∆*alaE*, MG1655∆*dadA*, and their parent MG1655 in a coculture system. As shown in Figure 2, MG1655 and MG1655∆*dadA* grew to nearly the same level after incubation in the presence of 5 mM Ala-Ala. In contrast, MG1655 and MG1655∆*dadA*, which have functional AlaE, outgrew MG1655∆*alaE* when they cocultured with MG1655∆*alaE* in the presence of Ala-Ala, indicating that the function of AlaE to extrude L-alanine is advantageous to cells under the conditions tested. 

The animal intestine, a natural habitat of *E. coli*, fluctuates between feast and famine environments for bacteria [31]. The concentration of Ala-Ala (5 mM) used in the coculture assay is physiologically relevant since alanine can occur at approximately 1.9 mM and 10.9 mM in its free- and peptide-forms, respectively, in the human intestine [31]. Furthermore, expression of the *alaE* gene has been recently found to be positively regulated by the global regulator Lrp in the presence of L-alanine [28]. In addition, Lrp is known to repress a high-affinity branched-chain amino acid importer LIV-I, which imports L-alanine, in the presence of L-alanine [3]. This regulatory network mediated by Lrp could contribute to the maintenance of the intracellular homeostasis of L-alanine. We, therefore, concluded that the L-alanine export activity of AlaE is of primary importance for *E. coli* cells to survive in the animal intestine, and it functions as a ‘safety-valve’ to prevent a toxic level accumulation of intracellular L-alanine. The AlaE function as a ‘safety valve’ to L-alanine seems to be L-alanine specific, since *alaE*-deficient mutant derived from MLA301 has exhibited a higher level of intracellular L-alanine than MLA301 in the presence of 1 mM of alanine-containing dipeptide, L-alanyl-glycine, L-alanyl-L-leucine, and L-alanyl-L-phenylalanine; however, levels of glycine, L-leucine, and L-phenylalanine were similar in both strains [20]. This function of AlaE is in good agreement with an earlier study in which *Corynebacterium glutamicum* was shown to possess a lysine-specific export system [32]. Furthermore, orthologs with high homology to AlaE have been found to present in very restricted bacterial groups, mainly in enteric bacteria [27].

Taken together, we concluded that the L-alanine exporter AlaE plays a critical role in avoiding a toxic accumulation of L-alanine and functions as the ‘safety valve’ under potential high L-alanine environments, such as in animal intestines.

## 3. Materials and Methods

### 3.1. Bacterial Strains, Plasmids, and Culture Conditions

The strains and plasmids used in this study are described in Table 2. *E. coli* cells were grown at 37 °C in L-broth containing 1% tryptone, 0.5% yeast extract, and 0.5% NaCl (pH 7.2) or minimal medium containing 22 mM glucose, 7.5 mM (NH_4_)_2_SO_4_, 1.7 mM MgSO_4_, 7 mM K_2_SO_4_, 22 mM NaCl, and 100 mM sodium phosphate (pH 7.1) [33]. When required, kanamycin (KM, 12.5 µg/mL), chloramphenicol (25 µg/mL), and ampicillin (50 µg/mL) were added to the medium. Growth was monitored by measuring the absorbance at 660 nm (A_660_).

### 3.2. Construction of a Single Mutant Lacking dadA and Double Mutants Lacking both alaE and dadA

To construct a single mutant lacking the *dadA* gene (MG1655∆*dadA*), we first amplified a DNA fragment containing a KM-resistant marker in the *dadA* gene using *dadA*-Fwd (5′-CTGGATAAAAAGGGCGTTCA-3′) and *dadA*-Rev (5′-CGTAAGCGTTCGCTTTTACC-3′) primers, a chromosomal DNA of JW1178 that lacks *dadA* as a template, and PrimeSTAR Max DNA polymerase (Takara, Shiga, Japan). The resulting DNA fragment was then transformed into MG1655 harboring pKD46 [36], and the transformants were selected on an L-agar plate supplemented with 12.5 µg/mL KM. To construct a double mutant lacking both the *alaE* and *dadA* genes (MG1655∆*dadA∆alaE*), a KM-resistant cassette inserted in the *dadA* gene of MG1655∆*dadA* was removed by transforming pCP20 [35]. Next, a DNA fragment containing a KM-resistant marker in the *alaE* gene was amplified by using *alaE*-Fwd (5′-TTACGGAATATTTTATCTAC-3′) and *alaE*-Rev (5′-ATGGCTTTAGTTCGCGTG-3′) primers, using the chromosomal DNA of the *alaE*-deficient strain JW2645 as a template. The resulting DNA fragments were then transformed into MG1655∆*dadA* to obtain the double mutant, as described above.

### 3.3. Susceptibility of the Mutants to L-alanyl-L-alanine (Ala-Ala)

The minimum inhibitory concentrations (MICs) of Ala-Ala for each strain were determined using the agar dilution method on minimal medium, as described by Kim et al. [29]. 

### 3.4. Determination of Intracellular Amino Acid Concentration

To determine intracellular amino acid concentration, cells (0.25 mL) grown in 4 mL of minimal medium with (for MLA301) or without (for MG1655 and its derivatives) 50 µg/mL of both D- and L-alanine at 37 °C overnight were inoculated into 25 mL of respective minimal medium and cultured to a mid-log phase. Cells were then collected by centrifugation (8900× *g*, 4 °C, 10 min) and washed twice with ice-cold minimal medium and suspended in pre-warmed (37 °C) minimal medium to give an A_660_ of 3. After a 10 min pre-incubation at 37 °C, the reaction was initiated by adding 6 mM Ala-Ala. Separation of the intracellular fractions was performed by the silicone oil method [27], in which the cells were placed onto the upper layer of a mixture of silicone oil KF-53 and KF-54 (8:2) (Shin-Etsu Chemical Co., Tokyo, Japan) with the lower layer consisting of 20% (*v/v*) perchloric acid, followed by centrifugation (20,000× *g*, 23 °C, 1 min). After removal of the aqueous phase and the silicone oil layer, cell pellets were sonicated in a bath-type sonicator (15 s, 23 °C), and the resulting cell suspension was centrifuged (20,000× *g*, 23 °C, 5 min). The supernatant was then neutralized with 2 M Na_2_CO_3_ to obtain the intracellular fraction. Amino acids in each fraction were quantified as their *o*-phthalaldehyde derivatives by a cation exchange column (Shim-pack AMINO NA; Shimadzu, Kyoto, Japan), with a high-performance liquid chromatography system (LC-10A; Shimadzu, Kyoto, Japan). To calculate the intracellular amino acid concentration, the intracellular volume was assumed to be 2.03 µL/mg dry weight per cell [37].

### 3.5. Coculture Assay

An approximately equal number of overnight culture cells of MG1655, MG1655∆*dadA*, and MG1655∆*alaE*, which harbored pBR322 or pBR325, were mixed in the combinations of MG1655(pBR322)/MG1655∆*dadA*(pBR325), MG1655(pBR322)/MG1655∆*alaE*(pBR325), and MG1655∆*dadA*(pBR322)/MG1655∆*alaE*(pBR325) in minimal medium containing 5 mM of Ala-Ala and 50 µg/mL of ampicillin. After growth to a late-log phase at 37 °C, cells were diluted to 10^−4^ to 10^−7^ with 0.85% NaCl, then each diluent was plated onto L-agar containing 25 µg/mL chloramphenicol or 50 µg/mL ampicillin and incubated at 37 °C overnight. The numbers of viable cells were determined after 18 h of incubation at 37 °C. Colony-forming units (CFU) containing pBR325 were determined by counting colonies grown on chloramphenicol-containing plates. The total CFU from each coculture experiment was determined by counting colonies grown on ampicillin plates. The CFU harboring pBR322 was then calculated by subtracting the CFU obtained on chloramphenicol plates from the total CFU.

## Figures and Tables

**Table 1:** MICs (minimum inhibitory concentrations) of L-alanyl-l-alanine in MG1655 and mutants.

	MIC (mg mL ^−1^)	
Strains	Glucose	Glycerol
MG1655	>1.25	>1.25
MG1655∆*dadA*	>1.25	>1.25
MG1655∆*alaE*	0.04	0.16
MG1655∆*dadA*∆*alaE*	0.0025	0.0025
MLA301	>1.25	>1.25
MLA301∆*alaE*	0.0025	0.0025

**Table 2:** Bacterial strains and plasmids used.

Strain or Plasmid	Characteristics	Reference
Strain		
*E. coli* MG1655	Wild type	Laboratory strain
*E. coli* JW1178	*dadA* disruptant derived from BW25113, KM^r^	[34]
*E. coli* JW2645	*alaE* disruptant derived from BW25113, KM^r^	[34]
*E. coli* MG1655 *alaE*	*alaE* disruptant derived from MG1655, KM^r^	[29]
*E. coli* MG1655 *dadA*	*dadA* disruptant derived from MG1655, KM^r^	This study
*E. coli* MG1655 *dadA* *alaE*	MG1655 *dadA* derivative with a deletion in the *alaE* gene, KM^r^	This study
*E. coli* MLA301	MG1655 *alr*::FRT, *dadX*::FRT, *yfdZ*::FRT, *avtA*::GM, *yfbQ*::KM	[20]
*E. coli* MLA301 *alaE*	MLA301 derivative with a deletion in the *alaE* gene, GM^r^, KM^r^	[27]
Plasmid		
pCP20	FLP^+^, λcI857^−^, λ*p*_R_Rep^ts^, Amp^r^, CP^r^	[35]
pKD46	Red recombinase expression plasmid	[36]
pBR322	Amp^r^, TET^r^, cloning vector	[27]
pBR325	Amp^r^, TET^r^, CP^r^, cloning vector	Laboratory stock
